# Risk of serious bacterial infections in inflammatory rheumatic or bowel disease patients during biological therapies: nationwide Danish cohort study

**DOI:** 10.1080/07853890.2025.2522968

**Published:** 2025-06-25

**Authors:** Emma Cathrine Højberg Platz, Gustav Emil Laugesen, Anne Ahrens Østergaard, Keld-Erik Byg, Inge Petersen, Isik Somuncu Johansen

**Affiliations:** a Department of Infectious Diseases, Odense University Hospital, Odense, Denmark; b Research Unit of Infectious Diseases, Department of Clinical Research, University of Southern Denmark, Odense, Denmark; c Department of Rheumatology, Odense University Hospital, Odense, Denmark; d Research Unit of Rheumatology, Department of Clinical Research, University of Southern Denmark, Odense, Denmark

**Keywords:** bDMARDs, anti-TNF alpha, bacterial infections, IBD, RA

## Abstract

**Introduction:**

To estimate the incidence rate ratio (IRR) of serious bacterial infections (SI) in patients with inflammatory bowel disease (IBD) or inflammatory rheumatic disease (IRD) before and after initiating biological therapy, and to assess comorbidities, infection sites, and the type of biological therapy.

**Methods:**

This nationwide retrospective registry-based study used a cohort-crossover design to assess the relative risk of SI one year before and after treatment. Data from the Danish National Patient Registry included 20,216 patients with IBD or IRD. Diagnosis codes for hospital contacts (inpatients, outpatients, day patients, and emergency department patients) and hospital-treated bacterial infections from 1994 to 2018 were linked using the Civil Registration System.

**Results:**

There were 1235 infections during 19,529 person-years one year before treatment, compared to 1251 infections over 17,831 person-years one year after treatment initiation (IRR = 1.11, 95% CI [1.03–1.19], *p* = 0.01). The incidence rate was 2-fold higher in the first three months post-treatment compared to over one year. Increased risks were found for bacterial infections without specified site; ear, nose, and throat; and respiratory tract infections. Higher risks of SI after treatment initiation were associated with comorbidities, treatment initiation between 2015 and 2018, age ≥61 at inflammatory disease diagnosis [HR = 1.50 (1.31–1.72)], female sex, and corticosteroid co-administration. Anakinra showed a significantly increased risk of infection [HR = 2.02, 95%CI 1.03–3.97)] in IRD patients.

**Conclusions:**

We found a modestly increased risk of SI in the year following therapy initiation, with the highest risk in the first three months. This risk was associated with certain baseline factors, highlighting the need for thorough risk assessment when starting treatment.

## Introduction

Treatment for inflammatory bowel diseases (IBD) and inflammatory rheumatic diseases (IRD) has been revolutionized by the introduction of biological therapy in Denmark in 1999 [1]. Since the introduction of biological therapy, its use has increased steadily, with an estimated 4000 IBD patients with ulcerative colitis (UC) and Crohn’s disease (CD), and 8481 IRD patients with rheumatoid arthritis (RA), psoriatic arthritis (PsA), spondyloarthropathy (SpA), and juvenile arthritis (JA) treated with biological therapy in Denmark in 2016 [2–6].

Biological therapies target specific molecules, such as tumor necrosis factor-alpha (TNF-α) and various interleukins (IL) since they are often involved in the inflammatory cascade of inflammatory diseases [7]. TNF-α and ILs play a pivotal role in the pathogenesis of various inflammatory disorders through complex signaling cascades [8,9]. Clinically approved TNF-α inhibitors include infliximab, adalimumab, and etanercept and IL inhibitors, such as anakinra (anti-IL1), tocilizumab (anti-IL6R), and ustekinumab (anti-IL12/IL23p40) are utilized to reduce disease activity in inflammatory diseases [9]. A limitation of TNF-α and IL inhibitors is that they are immunosuppressive and make the host vulnerable to infections [7].

Two retrospective studies assessed the risk of developing serious infections in IBD and IRD patients, identifying susceptibility, particularly to respiratory, gastrointestinal, and urinary tract infections following biological agents [10,11]. A recent meta-analysis of randomized controlled trials in IBD patients receiving IL inhibitors found no significant difference in infection risk compared to controls [12]. However, infection risk varied between TNF-α inhibitors and IL-inhibitors.

The overall risk is influenced by multiple factors, including patient age, underlying disease type and severity, specific biological therapy, and concomitant treatment with non-biologic immunosuppressive drugs [12–16]. Thus, initiating biological therapy requires careful assessment of the risk of inadequate disease control and the availability of alternative treatments [13]. However, previous studies have primarily focused on TNF-α inhibitors, relied on local or single-center cohorts, lacked control groups, or had limited follow-up periods, reducing generalizability. Additionally, the lack of robust evidence linking specific infections to particular inflammatory diseases complicates treatment decisions and contributes to uncertainties regarding the risk of serious bacterial infections (SI) in these patient populations.

Our objective was to estimate the incidence rate ratio of SI among patients diagnosed with IBD or IRD before and after the initiation of biological therapy. Furthermore, we sought to delineate the patient risk profiles according to factors, such as comorbidities, infection sites, and type of biological therapy administered.

## Methods

### Study design

This retrospective registry-based study included Danish residents diagnosed with IBD or IRD who were treated with biological therapy. A cohort-crossover design was conducted to assess the relative risk of SI before and after the introduction of treatment with biological therapies.

### Data sources

We included data on all Danish residents from the Danish National Patient Registry (DNPR) on diagnosis codes of IBD (CD and UC) and IRD (RA, JA, PsA, and SpA) based on the International Classification of Diseases, 10th revision (ICD-10) between 1994 and 2018 (Supplementary Table S1) [17,18].

From the DNPR, we obtained diagnosis codes regarding hospital contacts including inpatients, outpatients, day patients, and emergency department patients, and hospital-treated bacterial infections between 1994 and 2018, as well as comorbidities five years prior to IBD and IRD diagnosis and five years prior to introduction of biological therapy (Supplementary Table S1). Smoking data were not available in the registries.

The procedure codes from the DNPR regarding biological therapies administered at the hospital were obtained between 2002 and 2018. We linked the data by the personal identification numbers (PIN) from the Danish Civil Registration System (DCRS) that includes individual information on name, sex, citizenship, and date of birth [19]. The DCRS contains all Danish residents with a PIN, a unique 10-digit number, which is assigned to all Danish residents at birth or emigration, enabling linkage across Danish administrative registers. We excluded patients with an invalid PIN from the analysis, as the data could not be linked. We had remote online access to all data *via* the Secure Research Platform, managed by the Danish Health Data Authority’s.

### Study variables

Data on the specific biological drugs, corticosteroid, and immunomodulators administered are shown in Supplementary Table S2. A patient was classified as a user of biological therapy following the assignment of a relevant procedure- and ATC-code (Anatomical Therapeutic Chemical Classification System) for such treatment. The patient was considered unexposed six months after the last treatment. We did not account for prior immunosuppressive treatment before initiation of biologic therapy.

The primary outcome of the study was the incidence of SI, defined as a bacterial infection requiring hospital contact. Both primary and secondary diagnoses were included. The diagnostic codes for bacterial infections were categorized into 13 groups of site-specific infections in accordance with The Danish Medical Coding Classification System (Supplementary Table S1) [20]. If multiple diagnoses in the same subgroup occurred within 30 days of each other, they were regarded as a singular incidence, and the date of incidence was set to the first contact date within these clusters.

### Ethics

The study is conducted according to the Helsinki Declaration. Ethical approval is not required for registry-based studies in Denmark. Access to national health registry data for this study was approved by The Regional Committees on Health Research Ethics for Southern Denmark, which waived the requirement for informed consent. The study is approved by the Danish Data Protection Agency (Jnr. 19/8278) and the Danish Health Authority (Jnr: 31-1521-77). The Danish Data Protection Agency ensures compliance with the General Data Protection Regulation (GDPR) and national data protection laws. Approval from the Danish Health Authority permits access to data from national health registries in accordance with the study protocol, without requiring individual patients consent.

### Statistical analyses

#### Patients

Counts and median statistics were used to compare IBD and IRD patients who received biological therapy with those who did not.

#### Main analysis

The incidence rate ratio (IRR) for SI was calculated by comparing the incidence rate (IR) within one year after the initiation of biological therapy to the IR in the year preceding therapy initiation. The follow-up time was defined from either one year before or the last infection prior to treatment (whichever occurred later) to the first infection after treatment or one year post-treatment (whichever occurred first). The analysis period was adjusted if the patients died or emigrated before completing one year of follow-up. The follow-up time was set to end six months after the last registered treatment date. To accommodate potential correlations from repeated measurements across the same patient, a 95% confidence interval (CI) was estimated using 1000 repetitions of bootstrapping while clustering on patient identifiers. The analysis was repeated across four age strata (≤20, 21–40, 41–60, and ≥61) years at treatment initiation. Lastly, we assessed the time from initiation of biological therapy to the first infection by estimating IRs across four time intervals; 0–3 months, 3–6 months, 6–12 months, and 1+ year. The analyses were repeated stratified for IBD and IRD (Supplementary Tables S3a,b).

**Table 3.** Multivariable Cox regression analysis of risk factors for serious bacterial infections after introduction of biological therapy in patients with inflammatory bowel disease or inflammatory rheumatic disease.

	Any infection		Any infection excluding urinary tract and genitals		Ear, nose, and throat		Respiratory tract		Gastrointestinal tract		Bone and soft tissue		Urinary tract and genitals		Skin		Bacterial infections without specified site	
	HR (95% CI)	*p*-Value	HR (95% CI)	*p*-Value	HR (95% CI)	*p*-Value	HR (95% CI)	*p*-Value	HR (95% CI)	*p*-Value	HR (95% CI)	*p*-Value	HR (95% CI)	*p*-Value	HR (95% CI)	*p*-Value	HR (95% CI)	*p*-Value
Sex																		
Male	0.83 (0.77–0.88)	<0.001	0.95 (0.89–1.02)	0.18	0.74 (0.65–0.85)	<0.001	1.01 (0.91–1.13)	0.82	0.88 (0.76–1.01)	0.07	1.01 (0.76–1.35)	0.92	0.38 (0.33–0.43)	<0.001	0.87 (0.77–0.97)	0.02	0.97 (0.83–1.14)	0.75
Treatment year																		
2002–2009	1 (ref.)		1 (ref.)		1 (ref.)		1 (ref.)		1 (ref.)		1 (ref.)		1 (ref.)		1 (ref.)		1 (ref.)	
2010–2014	1.08 (1.00–1.16)	0.05	1.12 (1.03–1.21)	<0.01	1.51 (1.29–1.75)	<0.001	1.14 (1.01–1.28)	0.04	1.00 (0.84–1.19)	1.00	0.78 (0.57–1.07)	0.13	0.91 (0.81–1.03)	0.14	1.01 (0.89–1.16)	0.83	1.01 (0.84–1.21)	0.94
2015–2018	1.33 (1.21–1.47)	<0.001	1.36 (1.23–1.51)	<0.001	1.55 (1.27–1.90)	<0.001	1.23 (1.04–1.45)	0.01	1.07 (0.86–1.34)	0.53	0.55 (0.32–0.96)	0.03	0.87 (0.73–1.04)	0.13	0.88 (0.73–1.07)	0.20	1.25 (0.97–1.59)	0.08
Age at treatment																		
≤20	1 (ref.)		1 (ref.)		1 (ref.)		1 (ref.)		1 (ref.)		1 (ref.)		1 (ref.)		1 (ref.)		1 (ref.)	
21–40	0.97 (0.86–1.09)	0.58	0.97 (0.85–1.09)	0.60	0.68 (0.56–0.82)	<0.001	1.55 (1.18–2.03)	<0.01	1.24 (0.97–1.58)	0.09	1.04 (0.54–2.01)	0.90	0.89 (0.74–1.09)	0.26	1.03 (0.84–1.27)	0.77	1.30 (0.93–1.80)	0.12
41–60	1.03 (0.91–1.16)	0.63	1.05 (0.92–1.19)	0.51	0.49 (0.39–0.61)	<0.001	2.90 (2.20–3.82)	<0.001	1.05 (0.81–1.37)	0.71	1.48 (0.75–2.91)	0.26	0.99 (0.80–1.21)	0.89	1.06 (0.85–1.31)	0.63	1.90 (1.36–2.66)	<0.001
≥61	1.50 (1.31–1.72)	<0.001	1.47 (1.27–1.71)	<0.001	0.36 (0.26–0.48)	<0.001	6.08 (4.57–8.08)	<0.001	1.64 (1.21–2.23)	<0.01	1.66 (0.81–3.44)	0.17	1.97 (1.57–2.47)	<0.001	1.23 (0.96–1.59)	0.10	3.42 (2.39–4.89)	<0.001
Diagnosis causing treatment																		
Crohn’s disease	1 (ref.)		1 (ref.)		1 (ref.)		1 (ref.)		1 (ref.)		1 (ref.)		1 (ref.)		1 (ref.)		1 (ref.)	
Juvenile arthritis	0.86 (0.74–1.00)	0.05	0.91 (0.77–1.08)	0.27	1.20 (0.94–1.51)	0.14	1.22 (0.88–1.70)	0.23	0.41 (0.27–0.61)	<0.001	3.11 (1.48–6.57)	<0.01	0.62 (0.47–0.81)	<0.001	0.64 (0.48–0.86)	<0.01	0.44 (0.26–0.74)	<0.01
Psoriatic arthritis	0.73 (0.65–0.81)	<0.001	0.74 (0.65–0.83)	<0.001	0.81 (0.64–1.02)	0.08	0.71 (0.58–0.86)	<0.001	0.39 (0.29–0.51)	<0.001	1.66 (0.91–3.01)	0.10	0.55 (0.45–0.67)	<0.001	0.59 (0.48–0.72)	<0.001	0.48 (0.36–0.63)	<0.001
Rheumatoid arthritis	0.76 (0.69–0.84)	<0.001	0.80 (0.73–0.89)	<0.001	0.76 (0.62–0.94)	<0.01	1.05 (0.90–1.23)	0.53	0.36 (0.29–0.45)	<0.001	2.54 (1.51–4.28)	<0.001	0.52 (0.44–0.60)	<0.001	0.65 (0.55–0.76)	<0.001	0.57 (0.45–0.70)	<0.001
Spondyloarthropathy	0.60 (0.53–0.67)	<0.001	0.61 (0.54–0.68)	<0.001	0.88 (0.71–1.09)	0.23	0.64 (0.53–0.79)	<0.001	0.35 (0.27–0.45)	<0.001	1.28 (0.70–2.35)	0.42	0.50 (0.41–0.62)	<0.001	0.50 (0.41–0.62)	<0.001	0.32 (0.23–0.43)	<0.001
Ulcerative colitis	0.90 (0.81–0.99)	0.04	0.90 (0.81–1.01)	0.08	1.01 (0.82–1.23)	0.95	0.97 (0.80–1.17)	0.73	1.28 (1.06–1.54)	0.01	1.50 (0.79–2.86)	0.21	0.91 (0.77–1.08)	0.27	0.84 (0.70–1.01)	0.07	0.89 (0.70–1.13)	0.32
Comorbidities																		
Myocardial infarct	1.70 (1.11–2.59)	0.01	1.63 (1.03–2.57)	0.04	0.97 (0.24–3.97)	0.96	1.66 (0.95–2.92)	0.08	0.65 (0.19–2.23)	0.49	3.91 (1.26–12.08)	0.02	1.13 (0.53–2.40)	0.76	1.14 (0.53–2.46)	0.75	1.30 (0.53–3.19)	0.56
Congestive heart failure	1.22 (0.76–1.95)	0.41	1.14 (0.68–1.92)	0.61	0.45 (0.06–3.29)	0.43	0.65 (0.31–1.38)	0.26	2.35 (0.96–5.77)	0.06	1.66 (0.33–8.21)	0.54	1.40 (0.66–3.01)	0.38	2.39 (1.20–4.79)	0.01	1.14 (0.43–3.03)	0.79
Peripheral vascular disease	1.23 (0.61–2.47)	0.56	0.69 (0.26–1.83)	0.45	NA	.	0.27 (0.04–1.95)	0.20	0.89 (0.12–6.35)	0.91	NA	.	2.75 (1.22–6.18)	0.01	1.83 (0.59–5.70)	0.30	1.61 (0.40–6.53)	0.50
Cerebrovascular disease	1.09 (0.73–1.63)	0.68	0.96 (0.60–1.52)	0.85	1.45 (0.60–3.50)	0.41	1.12 (0.63–1.98)	0.70	1.54 (0.68–3.51)	0.30	2.46 (0.77–7.85)	0.13	1.85 (1.10–3.11)	0.02	0.61 (0.23–1.62)	0.32	0.60 (0.19–1.89)	0.38
Chronic pulmonary disease	1.75 (1.46–2.11)	<0.001	1.69 (1.38–2.07)	<0.001	1.95 (1.39–2.72)	<0.001	2.49 (1.91–3.23)	<0.001	1.66 (1.08–2.56)	0.02	0.45 (0.11–1.92)	0.28	1.54 (1.11–2.13)	0.01	1.11 (0.74–1.66)	0.60	2.03 (1.34–3.08)	<0.001
Hemiplegia or paraplegia	0.75 (0.11–5.32)	0.77	0.88 (0.12–6.26)	0.90	3.39 (0.48–24.13)	0.22	NA	.	NA	.	NA	.	NA	.	NA	.	NA	.
Renal disease	2.82 (1.50–5.31)	<0.01	2.92 (1.50–5.69)	<0.01	1.49 (0.21–10.64)	0.69	3.59 (1.46–8.82)	<0.01	3.81 (1.20–12.07)	0.02	NA	.	4.68 (1.91–11.49)	<0.001	1.56 (0.38–6.34)	0.53	5.45 (1.97–15.08)	<0.01
Diabetes	1.34 (1.04–1.73)	0.02	1.25 (0.94–1.65)	0.12	0.54 (0.22–1.30)	0.17	1.37 (0.95–1.97)	0.09	1.94 (1.16–3.23)	0.01	0.55 (0.13–2.29)	0.41	2.37 (1.67–3.35)	<0.001	2.17 (1.49–3.16)	<0.001	2.12 (1.33–3.40)	<0.01
Liver disease	0.89 (0.57–1.40)	0.61	0.99 (0.61–1.59)	0.96	1.86 (0.88–3.92)	0.10	0.57 (0.24–1.37)	0.21	NA	.	3.25 (1.04–10.20)	0.04	0.76 (0.34–1.70)	0.50	0.87 (0.36–2.09)	0.75	1.04 (0.39–2.81)	0.93
Cancer	1.43 (0.96–2.14)	0.08	1.58 (1.04–2.41)	0.03	1.17 (0.37–3.65)	0.79	1.44 (0.83–2.50)	0.19	0.99 (0.32–3.10)	0.99	2.05 (0.50–8.42)	0.32	0.75 (0.31–1.82)	0.53	1.66 (0.82–3.34)	0.16	1.44 (0.59–3.49)	0.42

Results are presented as hazard ratios (HR) for any infection, any infection excluding urinary tract and genitals, seven site-specific subgroups.

#### Comorbidities and other risk factors

Risk factors for SI (first infection, only) after introduction of biological therapy were identified using multivariable Cox regressions. This was done for any infection, any infection excluding urinary tract and genitals, and 7 out of the 13 site-specific infection groups. Infection sites with *n* < 100 cases were not included in this analysis due to insufficient data. Patients were censored at death, emigration, or six months after the last registered treatment. The analyzed risk factors were sex, age when treatment was introduced (categorized into four groups), treatment year, inflammatory disease causing biological therapy, and comorbidities five years prior to the start of treatment. Three comorbidity groups were pooled: (1) diabetes with and without organ damages, (2) mild and severe liver diseases, and (3) malignancies and solid tumors. Results are presented as Hazard Ratios (HR).

#### Type of biological therapy

Using Cox regression a HR was calculated to estimate the risk of SI due to the various biological therapies. The patient’s timeline was segmented at each change in treatment. Infliximab was used as reference due to being the most frequently administered drug. If a patient received multiple registration of biological therapy on the same day, they were classified under a ‘Combined’ section. The analysis was further stratified for patients treated for IBD and IRD. However, certain biological therapies were rarely used in IBD patients and were therefore excluded from the analysis. Additionally, the effect of co-administration of corticosteroid and immunomodulators was assessed. This included treatments administered from 90 days before the initiation or change of a biological agent to 14 days after. These analyses focused on the most frequently used biological therapies and were performed using Cox regression.

All analyses were adjusted for age (continuous) at treatment initiation, changes in biological therapy, and sex. Robust variance estimators were used to address the repeated measurements within patient correlations.

All statistical analyses were performed using Stata 18 [21]. The statistical results were considered statistically significant if *p*-values did not exceed 0.05.

## Results

### Participants

**Figure 1.**
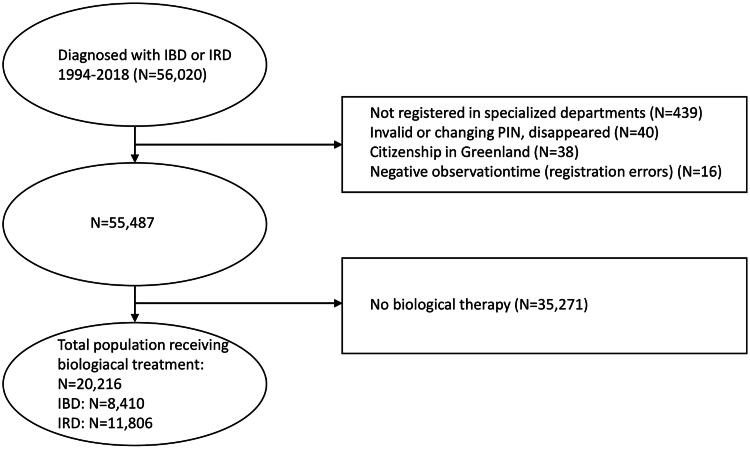
Total study population with diagnoses of inflammatory bowel disease (IBD) or inflammatory rheumatic disease (IRD) treated with biological therapy. PIN: personal identification numbers; IBD: inflammatory bowel disease; IRD: inflammatory rheumatic disease.

Between 1994 and 2018, 56,020 patients were identified with diagnoses of IRD or IBD. Of these, 35,271 (62.96%) were excluded from further analyses as they did not receive biological therapy. A total of 20,216 (36.1%) patients with IRD or IBD were included in the analyses following the exclusion criteria outlined in Figure 1. Of the 20,216 patients, 41.6% had IBD, 42.3% were male, and the median age at onset of inflammatory disease was 36 years. The median age was significantly higher in patients with IRD compared to those with IBD (47 [IQR = 34–58] *vs.* 33 years [IQR = 23–46], *p*-value <0.05). Baseline characteristics of patients by treatment status are presented in Table 1.

**Table 1.** Baseline characteristics of patients with inflammatory bowel disease or inflammatory rheumatic disease.

Characteristics	Biological therapy (*N* = 20,216)	No biological therapy (*N* = 35,271)
Age at inflammatory disease diagnosis	36 (23–49)	44 (30–57)
Diagnosed before 1999 (*N* = 9332)		
≤20 years	587 (50.6%)	572 (49.4%)
21–40 years	1701 (37.6%)	2820 (62.4%)
41–60 years	1133 (33.5%)	2251 (66.5%)
≥61 years	58 (21.6%)	210 (78.4%)
Diagnosed between 2000 and 2004 (*N* = 6479)		
≤20 years	466 (50.9%)	450 (49.1%)
21–40 years	1106 (42.5%)	1499 (57.5%)
41–60 years	928 (38.6%)	1478 (61.4%)
≥61 years	132 (23.9%)	420 (76.1%)
Diagnosed between 2005 and 2009 (*N* = 9888)		
≤20 years	800 (61.4%)	503 (38.6%)
21–40 years	1684 (51.2%)	1608 (48.8%)
41–60 years	1655 (43.3%)	2164 (56.7%)
≥61 years	403 (27.3%)	1071 (72.7%)
Diagnosed between 2010 and 2014 (*N* = 14,120)		
≤20 years	1146 (57.2%)	859 (42.8%)
21–40 years	2062 (45.5%)	2471 (54.5%)
41–60 years	1784 (36.7%)	3072 (63.3%)
≥61 years	592 (21.7%)	2134 (78.3%)
Diagnosed between 2015 and 2018 (*N* = 15,668)		
≤20 years	777 (37.8%)	1276 (62.2%)
21–40 years	1571 (31.6%)	3399 (68.4%)
41–60 years	1139 (22.8%)	3856 (77.2%)
≥61 years	492 (13.5%)	3158 (86.5%)
Sex		
Male	8547 (36.8%)	14,696 (63.2%)
Diagnosis		
Crohn’s disease	4577 (45.4%)	5497 (54.6%)
Ulcerative colitis	3833 (20.7%)	14,648 (79.3%)
Rheumatoid arthritis	5411 (39.0%)	8472 (61.0%)
Juvenile arthritis	1128 (47.3%)	1259 (52.7%)
Spondyloarthropathy	2871 (61.4%)	1808 (38.6%)
Psoriatic arthritis	2396 (40.0%)	3587 (60.0%)
Comorbidities		
Myocardial infarct	114 (30.4%)	261 (69.6%)
Congestive heart failure	80 (25.9%)	229 (74.1%)
Peripheral vascular disease	53 (22.0%)	188 (78.0%)
Cerebrovascular disease	146 (23.2%)	482 (76.8%)
Chronic pulmonary disease	613 (34.0%)	1188 (66.0%)
Mild liver disease	127 (33.6%)	251 (66.4%)
Moderate-severe liver disease	16 (42.1%)	22 (57.9%)
Diabetes without organ damage	347 (29.0%)	850 (71.0%)
Diabetes with organ damage	77 (32.4%)	161 (67.6%)
Hemiplegia or paraplegia	12 (24.5%)	37 (75.5%)
Renal disease	36 (23.5%)	117 (76.5%)
Malignancy	149 (16.2%)	771 (83.8%)
Solid tumor	14 (20.0%)	56 (80.0%)

### Main results

A total of 1235 infections were registered during 19,529 person-years one year before treatment with biological therapy compared to 1251 infections over 17,831 person-years one year after treatment initiation (any infection IRR = 1.11 [95% CI 1.03–1.19], *p*-value 0.01) (Table 2). The increased risk was primarily driven by biological therapy in IRD patients (IRR = 1.22 [95% CI 1.10–1.36], *p*-value <0.001), whereas no significant increase was detected in IBD patients (IRR = 1.02 [95% CI 0.92 − 1.13], *p*-value 0.77). The risk of SI was not increased in age groups ≤20 and 21–40 (IRR = 0.86 and 0.97, respectively). However, for the two oldest age groups, the IRR increased by ∼30% (IRR = 1.35 and 1.28, respectively) and this increase was statistical significant only among IRD patients. The highest statistically significant site-specific IRR was demonstrated in the category of bacterial infections without specified site (IRR = 1.47 [95% CI 1.13–1.92], *p*-value <0.01), ear, nose, and throat (ENT) (IRR = 1.32 [95% CI 1.09–1.60], *p*-value <0.01) and respiratory tract infections (IRR = 1.35 [95% CI 1.15–1.58], *p*-value <0.001). The IRR of SI is shown separately for IBD and IRD in Supplementary Tables S3a,b.

**Table 2.** Incidence rate ratios (IRR) of serious bacterial infections among patients with inflammatory bowel disease or inflammatory rheumatic disease one year before and after initiation of biological therapy.

	Total sample	*N* infections before treatment	Observation time before treatment (years)	*N* infections after treatment	Observation time after treatment (years)	IRR	*p*-Value
Any infection							
IRD/IBD	20,216	1235	19,529	1251	17,831	1.11 (1.03–1.19)	0.01
IRD	11,806	576	11,513	650	10,634	1.22 (1.10–1.36)	<0.001
IBD	8410	659	8016	601	7197	1.02 (0.92–1.13)	0.77
Any infection stratified by age							
−20	2604	216	2482	170	2281	0.86 (0.70–1.04)	0.13
21–40	7150	447	6898	400	6356	0.97 (0.85–1.10)	0.66
41–60	7524	349	7333	431	6730	1.35 (1.18–1.54)	<0.001
61+	2938	223	2817	250	2464	1.28 (1.08–1.52)	<0.01
Site specific infections							
Bacterial infections without specified site	20,216	102	20,158	137	18,406	1.47 (1.13–1.92)	<0.01
Central nervous system	20,216	<5	20,215	<5	18,466	2.19 (0.52–9.29)	0.29
Ear, nose, and throat	20,216	176	20,125	212	18,359	1.32 (1.09–1.60)	<0.01
Respiratory tract	20,216	268	20,077	330	18,305	1.35 (1.15–1.58)	<0.001
Spirochetes	20,216	<5	20,215	6	18,466	2.19 (0.54–8.84)	0.27
Zoonotic bacterial infections	20,216	5	20,213	5	18,466	1.09 (0.29–4.08)	0.89
Heart	20,216	<5	20,214	<5	18,466	1.09 (0.25–4.73)	0.90
Skin	20,216	222	20,094	228	18,355	1.12 (0.93–1.35)	0.22
Urinary tract and genitals	20,216	271	20,069	247	18,347	1.00 (0.84–1.18)	0.97
Other infectious agents	20,216	271	20,057	209	18,361	0.84 (0.71–1.00)	0.05
Gastrointestinal tract	20,216	5	20,213	<5	18,467	0.44 (0.10–1.86)	0.26
Eyes	20,216	68	20,181	37	18,451	0.60 (0.40–0.89)	0.01
Bone and soft tissue	20,216	102	20,158	137	18,406	1.47 (1.13–1.92)	<0.01

**Figure 2.**
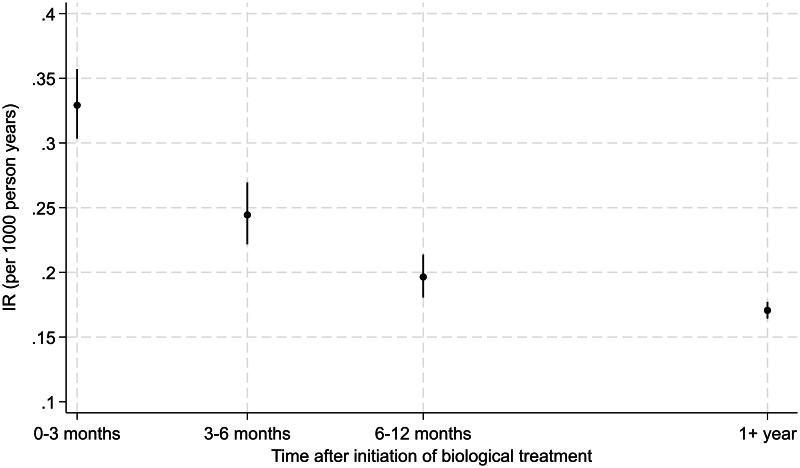
Incidence rates (IR) for serious bacterial infections per 1000 person-years after initiation of biological therapy in patients with inflammatory bowel disease or inflammatory rheumatic disease.

The IR was highest in the first three months following the initiation of biological therapy (IR = 0.33 [95% CI 0.31–0.36] per 1000 person-years) and subsequently decreased to its lowest point after one year (IR = 0.17 [95% CI 0.16–0.18] per 1000 person-years), equivalent to an IRR of 1.92 [95% CI 1.76–2.10] (Figure 2).

### Comorbidities and other risk factors

The results from the multivariable Cox regression analysis of risk factors are shown in Table 3. The risk of both site-specific and overall infections was generally higher in the presence of comorbidities. The HR for developing infections increased when initiating biological therapy at ≥61 years, especially for bacterial infections without specified site and respiratory tract infections. There was a significant increase in the HR in the treatment period between 2015 and 2018 for any infection (HR = 1.33 [95% CI 1.21–1.47], *p*-value <0.001). This trend also applied for ENT, respiratory tract, and bacterial infections without specified site. Conversely, being male was associated with a reduced risk of infections in urinary tract and genital infections (HR = 0.38 [95% CI 0.33–0.43], *p*-value <0.001) and in any infection (HR = 0.83 [95% CI 0.77–0.88], *p*-value <0.001).

### Differences in biological therapy

Results of the Cox regression analysis assessing the risk of infections associated with different biological therapies, using infliximab as the reference, stratified by IBD and IRD are shown in Table 4. Adalimumab (HR = 0.77 [95% CI 0.69–0.85], *p*-value <0.001), golimumab (HR = 0.79 [95% CI: 0.65–0.97], *p*-value 0.03) and etanercept (HR = 0.64 [95% CI 0.57–0.73], *p*-value <0.001) were associated with a significantly reduced risk of SI compared to infliximab. These associations were also observed in IRD patients, whereas the risk was not reduced for IBD patients. Conversely, anakinra (HR = 2.02 [95% CI 1.03–3.97], *p*-value 0.04) was associated with an increased risk of SI in IRD patients.

**Table 4.** Hazard ratios (HR) from a multivariable Cox regression, for serious bacterial infections due to various biological therapies, in patients with inflammatory bowel disease or inflammatory rheumatic disease.

Biological therapies	Total sample		IBD		IRD	
	HR (95% CI)	*p*-Value	HR (95% CI)	*p*-Value	HR (95% CI)	*p*-Value
Tumor necrosis factor alpha inhibitors, N*						
Infliximab (ref) *N* = 12.109	1 (ref)		1 (ref)		1 (ref)	
Adalimumab *N* = 7.259	0.77 (0.69–0.85)	<0.001	1.16 (0.99–1.35)	0.07	0.68 (0.59–0.78)	<0.001
Golimumab *N* = 1.909	0.79 (0.65–0.97)	0.03	1.27 (0.83–1.95)	0.27	0.79 (0.62–1.00)	0.05
Certolizumab pegol *N* = 1.771	0.82 (0.67–1.01)	0.06	1.46 (0.78–2.74)	0.24	0.85 (0.68–1.07)	0.17
Etanercept *N* = 5.666	0.64 (0.57–0.73)	<0.001	1.16 (0.65–2.06)	0.61	0.70 (0.60–0.81)	<0.001
Interleukin inhibitors						
Tocilizumab *N* = 1.405	0.85 (0.74–1.05)	0.13	NA		0.90 (0.72–1.13)	0.38
Anakinra *N* = 97	1.92 (1.04–3.55)	0.04	NA		2.02 (1.03–3.97)	0.04
Secukinumab *N* = 365	0.79 (0.41–1.53)	0.48	NA		0.89 (0.46–1.72)	0.72
Ustekinumab *N* = 310	1.00 (0.61–1.63)	1.00	1.15 (0.49–2.72)	0.75	1.03 (0.57–1.87)	0.92
Abatacept *N* = 844	0.96 (0.69–1.24)	0.74	NA		1.01 (0.77–1.33)	0.92
Combination of two biologics						
*N* = 1030	1.03 (0.63–1.68)	0.91	0.35 (0.05–2.57)	0.30	1.27 (0.76–2.10)	0.36
Adjusted for						
Male	0.78 (0.72–0.85)	<0.001	0.77 (0.68–0.88)	<0.001	0.80 (0.72–0.89)	<0.001
Age (continuous)	1.01 (1.00–1.01)	<0.001	1.01 (1.00–1.01)	0.01	1.01 (1.01–1.01)	<0.001

*Number of patients receiving at least once.

Patients exposed to corticosteroids in combination with infliximab (HR = 1.34 [95% CI 1.13–1.58], *p*-value <0.001), etanercept (HR = 1.47 [95% CI 1.07–2.02], *p*-value 0.02), and adalinmumab (HR = 1.35 [95% CI 1.04–1.75], *p*-value = 0.02) had an increased SI risk compared to those exposed to the respective biological agents alone. However, this association was not observed for other biologics or for biological agents used in combination with immunomodulators (Table 5).

**Table 5.** Effect modification by corticosteroid and immunomodulators.

	Steroids		Steroids/immunomodulators		Immunomodulators	
	HR (95%CI)	*p*-Value	HR (95%CI)	*p*-Value	HR (95%CI)	*p*-Value
Infliximab	1.34 (1.13–1.58)	<0.001	1.04 (0.86–1.25)	0.69	0.77 (0.65–0.91)	0.00
Etanercept	1.47 (1.07–2.02)	0.02	1.58 (1.15–2.19)	0.01	1.10 (0.85–1.42)	0.49
Adalimumab	1.35 (1.04–1.75)	0.02	1.16 (0.85–1.58)	0.34	0.97 (0.80–1.18)	0.76
Abatacept	1.22 (0.65–2.30)	0.53	1.23 (0.59–2.58)	0.58	0.44 (0.21–0.93)	0.03
Tocilizumab	1.39 (0.87–2.22)	0.17	1.00 (0.48–2.08)	1.00	0.91 (0.55–1.49)	0.70
Golimumab	1.08 (0.56–2.09)	0.82	1.30 (0.59–2.87)	0.51	0.84 (0.48–1.46)	0.54
Certolizumab pegol	1.48 (0.83–2.62)	0.18	1.55 (0.84–2.86)	0.16	1.31 (0.82–2.10)	0.25

## Discussion

This nationwide register-based study identified an 11% increased risk of SI in the first year after the initiation of biological therapy. This risk was primarily driven by patients with IRD. The IR was twice as high in the first three months compared to 1+ year. Site-specific analysis revealed an increased risk for bacterial infections at unspecified site, ENT, and respiratory tract infections, while the risk of bone and soft tissue infections was lower. Risk factors associated with a higher IRR included renal disease, initiation of biological therapy between 2015 and 2018, age ≥61, and female sex. Among biological therapies, anakinra was specifically associated with a statistically significant increased risk of infection in IRD patients. Finally, combination therapy with two biologics did not increase the risk of SI, whereas concomitant use of TNF-α inhibitors and corticosteroids was associated with an increased risk.

The strengths of this study derive from the use of the DNPR, which enabled the inclusion of the entire population of patients with IBD or IRD in Denmark, with minimal loss to follow-up and only 0.21% of the cohort excluded [17]. The DNPR provided comprehensive exposures and outcome data, enhancing the reliability of our findings. Previous validations demonstrated high completeness (94% for UC and CD) and validity (97% for CD and 90% for UC initially, later 82% for both) [22,23]. For RA, completeness was 90%, with a validity of 79%, compared to 92% in the Danish Rheumatologic Database (DAN-BIO) [24]. Although data from DAN-BIO were not used in this study, they represent a potential resource for future research.

Our cohort-crossover design limited confounding by indication and ensured similar characteristics in the study groups which helps mitigate both measured and unmeasured confounding [25,26]. Another strength lies in its methodological approach, where exposure was defined as the patient’s initial biological therapy, reducing prevalent user bias, by ­including only incident users [25]. Outcomes were restricted to SI requiring hospital contacts, excluding non-bacterial pathogens. Tuberculosis was excluded due to ­mandatory pretreatment screening and treatment protocols [27].

Our study has limitations inherent to registry-based data, including potential misclassification of exposures and outcomes. While exposure misclassification is minimal due to controlled treatment administration in healthcare facilities, outcome misclassification may affect result validity [25,26]. Additionally, the accuracy of specific bacterial infection data in the DNPR remains unverified, potentially influencing conclusions. Furthermore, not all biologic therapies are represented in our cohort, particularly newer agents, due to the study period and limited use. Finally, we did not adjust for multiple testing across the 13 site-specific infection outcomes.

Although DNPR data enabled adjustment for comorbidities, disease, progression and newly developed comorbidities over the five-year study period were unaccounted for. Similarly, unmeasured confounders like lifestyle factors, seasonality, and smoking habits were not considered.

Consistent with earlier research, our findings align with a Danish propensity score-matched cohort study of 1543 IBD patients treated with TNF-α inhibitors, which reported an increased risk of SI within the first 90 days of treatment (HR = 1.63, 95% CI 1.01–2.63) [28]. Similarly, a British study using the BSRBR registry found a higher risk of SI in RA patients treated with TNF-α inhibitors compared to non-biologic immunomodulators (HR = 1.2, 95% CI 1.1–1.5), peaking in the first six months (HR 1.8, 95% CI 1.3–2.6) [13]. A meta-analysis of nine randomized placebo-controlled trials in RA patients receiving infliximab or adalimumab (≥ 12 weeks) estimated a pooled OR of 2.0 (95% CI 1.3–3.1) for SI, with a number needed to harm of 59 (95% CI 39–125) over 3–12 months [29].

Conversely, a population-based cohort study from British Columbia of 10,662 IBD patients found no significant association between infliximab and SI risk (adjusted IRR 1.08, 95% CI 0.42–2.74) [30]. Notably, multiple studies, including ours, observed the highest SI risk within the initial treatment period (first 90 days to six months) [13,28]. We found the IR for SI peaked in the first three months, declining thereafter due to early infections among susceptible individuals and depletion of the high-risk population [25]. Increased clinical vigilance post-biological therapy initiation may also inflate the IR estimates. Additionally, we observed a higher IRR for SI in older patients, likely reflecting both age-related susceptibility and increased disease severity and comorbidities. The observed differences in SI risk between IRD and IBD patients in our study may be partly explained by the higher median age in the IRD group. These findings highlight the need for heightened infection surveillance in newly treated and elderly patients.

Our results align with an Italian retrospective cohort study of 5596 IRD patients, where 45.3% of hospitalizations for infection were respiratory (excluding tuberculosis), with elderly patients having a 4-fold increased risk [11]. Similarly, we found a strong association between age ≥61 years and respiratory tract infections (HR = 6.16, 95% CI 4.62–8.22), supporting targeted prevention strategies, such as pneumococcal vaccination before biological therapy initiation along with annual influenza and Covid-19 vaccination, as recommended by national guidelines [11,31,32].

Interestingly, male sex appeared protective against SI; however, this effect was driven by urinary tract and genital infections, to which females are more susceptible. When these infections were excluded, male sex was no longer a protective factor.

We found the increased risk of infection when biological agents were co-administered with corticosteroids, particularly for certolizumab pegol and etanercept as described previously [30,33]. Interestingly, the risk of infection generally decreased when biological agents were used in combination with immunomodulators without steroids, except in the case of certolizumab pegol and etanercept. Our finding of an increased SI risk of anakinra in IRD patients aligns with current Danish Medicines Council (DMC) guidelines, which do not recommend anakinra for RA due to its limited disease-modifying effect and potential for increased infection risk [34]. The guidelines do not differentiate between abatacept, adalimumab, certolizumab, etanercept, golimumab, infliximab, or tocilizumab, mainly prioritizing cost when efficacy is comparable. However, for infection-prone patients, DMC recommends agents with a shorter half-life, such as etanercept or adalimumab, supporting our findings. Giving varying recommendations for PsA, UC, and CD, disease-specific analyses of infection risk remain essential [35–37]. The modest SI risk related to biologics must be weighed against their therapeutics benefits, including diseases control and prevention of irreversible in IRD or IBD. While these results warrant cautious interpretation and further investigation, clinicians should consider etanercept or adalimumab over infliximab for infection susceptible patients when treatments are otherwise deemed equivalent.

The study cohort is representative of the relevant population. The Danish healthcare system’s universal access and minimal private sector involvement (which rarely treats SI or administers biological therapies) minimize selection bias [17]. Excluding only 0.21% of the cohort further enhances the external validity of this study [38]. While the results apply to the study population, combining IBD and IRD patients may limit individual applicability. With 20,216 patients included, the study has adequate statistical power [39]. However, a sample size this large can increase the likelihood of deeming clinically negligible differences statistically significant. Therefore, results should be interpreted with consideration of their clinical relevance [40].

## Conclusion

In this nationwide cohort study of IBD and IRD patients undergoing biological therapy, we observed a modestly increased risk of a serious bacterial infection, primarily in IRD patients, with the highest in the first three months post-treatment. The most prevalent infection sites were bacterial infections without specified site; ear, nose, and throat; and respiratory tract infections. Advanced age, comorbidities, and corticosteroid co-administration were key risk factors. These findings underscore the importance of careful risk assessment, close monitoring, and improved preventive strategies to mitigate infection risk in high-risk patients.

## Supplementary Material

Revised Supplementary.docx

## Data Availability

The Danish Data Protection Agency has approved this study, stipulating that the data used cannot be shared publicly. However, data can be accessed at our institution upon a reasonable request to the corresponding author.
