# Supplementary material for: Risk of serious bacterial infections in inflammatory rheumatic or bowel disease patients during biological therapies: nationwide Danish cohort study
Source: Ann Med. 2025 Jun 25;57(1):2522968. doi: 10.1080/07853890.2025.2522968 (PMC12931301; doi:10.1080/07853890.2025.2522968)
Supplement: Revised Supplementary.docx [file IANN_A_2522968_SM7278.docx]

Table S1. Diagnosis codes based on the International Classification of Diseases, 10th revision, for sample identification, infections, and comorbidities from the Danish National Patient Registry

| **Disease** | **International Classification of Diseases 10th Revision codes** |
| --- | --- |
| **Population** |  |
| Crohn’s Disease | DK50, DM074 |
| Ulcerative Colitis | DK51, DM075 |
| Rheumatoid Arthritis | DM05, DM06 |
| Juvenile Arthritis | DM08, DM09 |
| Spondyloarthropathy | DM45, DM46 |
| Psoriatic Arthritis | DM07, DL405 |
| **Site-specific bacterial infections (endpoints)** |  |
| Central nervous system | DA321, DA390, DG00, DG01, DG06, DG042, DG050 |
| Ear, nose, and throat | DA36, DA38, DH600-603, DH65, DH66, DH670, DH680, DH70, DH730, DH731, DJ01, DJ03-06, DJ32, DJ340, DJ369, DJ370, DJ390, DJ391 |
| Gastrointestinal tract | DA00-06, DK121, DK122, DK20, DK35-37, DK65, DK67, DK750, DK751, DK770, DK81 |
| Respiratory tract | DA310, DA310A-C, DA37, DA42, DA481, DA493, DJ13-18, DJ200-202, DJ209, DJ22, DJ40-42, DJ85, DJ86 |
| Eyes | DH100, DH105, DH440, DH451, DH000A, DH000B, DH000D |
| Bone and soft tissue | DK102, DM00, DM010, DM012, DM013, DM462-465, DM491, DM492, DM600, DM608, DM630, DM632, DM650, DM651, DM680, DM710, DM711, DM86, DM901 |
| Urinary tract and genitals | DA419A, DN10-12, DN300-303, DN308B, DN308C, DN308D, DN308E, DN308F, DN308G, DN308K, DN309, DN340, DN341, DN342A, DN342B, DN342C, DN390, DN41, DN45, DN481, DN482, DN49, DN61, DN70-74, DN751, DN76 |
| Heart | DI301A, DI301B, DI301C, DI301D, DI320, DI33, DI400, DI410, DI520 |
| Skin | DA311, DA311A-B, DA318, DA319, DA320, DA46, DA483, DL00-04, DL08 |
| Zoonotic bacterial infections | DA20-28 |
| Bacterial infections without specified site | DA327, DA329, DA359, DA39, DA391, DA392, DA392A, DA393, DA394, DA398, DA399, DA400, DA401, DA403, DA408, DA409, DA41, DA410, DA411, DA411A, DA412, DA413, DA414, DA415, DA415A, DA418, DA419, DA419A, DA419C, DA439, DA449, DA480, DA488, DA49, DA490, DA491, DA491A, DA492, DA493, DA498, DA499, DA499A, DT802D, DT814D, DB95, DB96 |
| Spirochetes | DA69 |
| **Comorbidities** |  |
| Myocardial infarct | DI21, DI22, DI252 |
| Congestive heart failure | DI099, DI110, DI130, DI132, DI255, DI420, DI425-429, DI43, DI50, DP290 |
| Peripheral vascular disease | DI70, DI71, DI731, DI738, DI739, DI771, DI790, DI792, DK551, DK559, DZ958, DZ959 |
| Cerebrovascular disease | DG45, DG46, DH340, DI60-69 |
| Chronic pulmonary disease | DI278, DI279, DJ43-DJ47, DJ60-67, DJ684, DJ701, DJ703 |
| Mild liver disease | DB18, DK700-703, DK709, DK713-715, DK717, DK73, DK74, DK760, DK762-764, DK768, DK769, DZ944 |
| Moderate-severe liver disease | DI850, DI859, DI864, DI982, DK704, DK711, DK721, DK729, DK765-767 |
| Diabetes without organ damage | DE100, DE101, DE106, DE108-111, DE116, DE118-121, DE126, DE128-131, DE136, DE138-141, DE146, DE148, DE149 |
| Diabetes with organ damage | DE102-105, DE107, DE112-115, DE117, DE122-125, DE127, DE132-135, DE137, DE142-145, DE147 |
| Hemiplegia or paraplegia | DG041, DG114, DG801, DG802, DG81, DG82, DG830-834, DG839 |
| Renal disease | DI120, DI131, DN032-037, DN052-057, DN18, DN19, DN250, DZ490-492, DZ940, DZ992 |
| Malignancy | DC00-26, DC30-34, DC37-41, DC43, DC45-58, DC60-76, DC81-85, DC88, DC90-97 |
| Solid tumor | DC77-80 |

Table S2: Procedure codes and Anatomical Therapeutic Chemical Classification System codes on biological therapies obtained from The Danish National Patient Registry, 2002-2018.

| **Biological therapies** | **Anatomical Therapeutic Chemical Classification System codes** | **Procedure code** |
| --- | --- | --- |
| Anakinra | ML04AC03 | BOHJ18B1 |
| Infliximab | ML04AB02, ML04AA12 | BOHJ18A1 |
| Etanercept | ML04AB01, ML04AA11 | BOHJ18A2 |
| Adalimumab | ML04AB04, ML04AA17 | BOHJ18A3 |
| Abatacept | ML04AA24 | BOHJ18C1 |
| Tocilizumab | ML04AC07 | BOHJ18B2 |
| Golimumab | ML04AB06 | BOHJ18A4 |
| Certolizumab pegol | ML04AB05 | BOHJ18A5 |
| Secukinumab | ML04AC10 | BOHJ18B5 |
| Ustekinumab | ML04AC05 | BOHJ18B3 |
| Canakinumab | ML04AC08 | BOHJ18B4 |
| Ixekizumab | ML04AC13 |  |
| Apremilast | ML04AA32 |  |
| Guselkumab | ML04AC16 | BOHJ18B7 |
| Baricitinib | ML04AA37 |  |
| Tofacitinib | ML04AA29 |  |
| Natalizumab | ML04AA23 |  |
| Tumor necrosis factor alpha antibodies |  | BOHJ18A |
| Biological anti-rheumatic drugs |  | BOHJ18 |
| **Steroids** |  |  |
|  | MH02AB |  |
|  | MR03BA |  |
| **Immunomodulators** |  |  |
| Methotrexate | ML04AX03 |  |
| Leflunomide | ML04AA13 |  |
| Hydroxychloroquine | MP01BA02 |  |
| Sulfasalazine | MA07EC01 |  |
| Azathioprine | ML04AX01 |  |
| Chloroquine | MP01BA01 |  |

**Supplementary table S3a:** Incidence rate ratios (IRR) of serious bacterial infections among patients with inflammatory bowel disease one year before and after initiation of biological therapy

|  | Total sample | N infections before treatment | Observation time before treatment (years) | N infections after treatment | Observation time after treatment (years) | IRR | p-value |
| --- | --- | --- | --- | --- | --- | --- | --- |
| **Any infection** |  |  |  |  |  |  |  |
|  | 8410 | 659 | 8016 | 601 | 7197 | 1.02 (0.92-1.13) | 0.77 |
| **Any infection stratified by age** |  |  |  |  |  |  |  |
| -20 | 1524 | 134 | 1445 | 101 | 1346 | 0.81 (0.63-1.04) | 0.09 |
| 21-40 | 3950 | 300 | 3775 | 262 | 3415 | 0.97 (0.82-1.13) | 0.67 |
| 41-60 | 2297 | 156 | 2201 | 168 | 1940 | 1.22 (1.00-1.49) | 0.05 |
| 61+ | 639 | 69 | 595 | 70 | 496 | 1.22 (0.89-1.66) | 0.22 |
| **Site specific infections** |  |  |  |  |  |  |  |
| Bacterial infections without specified site | 8410 | 55 | 8375 | 72 | 7466 | 1.47 (1.04-2.07) | 0.03 |
| Central nervous system | 8410 | <5 | 8409 | <5 | 7499 | 1.12 (0.33-3.76) | 0.85 |
| Ear, nose, and throat | 8410 | 86 | 8366 | 107 | 7447 | 1.40 (1.07-1.83) | 0.01 |
| Respiratory tract | 8410 | 82 | 8367 | 108 | 7449 | 1.48 (1.12-1.96) | 0.01 |
| Spirochetes | 8410 | <5 | 8410 | <5 | 7499 | 4.49 (1.06-19.05) | 0.04 |
| Zoonotic bacterial infections | 8410 | <5 | 8409 | <5 | 7499 | 1.12 (0.25-5.08) | 0.88 |
| Heart | 8410 | <5 | 8410 | <5 | 7500 | 2.24 (0.58-8.61) | 0.24 |
| Skin | 8410 | 126 | 8333 | 113 | 7446 | 1.00 (0.78-1.28) | 0.98 |
| Urinary tract and genitals | 8410 | 141 | 8325 | 115 | 7444 | 0.91 (0.71-1.17) | 0.47 |
| Gastrointestinal tract | 8410 | 225 | 8273 | 148 | 7423 | 0.73 (0.60-0.89) | <0.01 |
| Eyes |  |  |  |  |  | NA |  |
| Bone and soft tissue | 8410 | 8 | 8405 | 10 | 7496 | 1.40 (0.54-3.66) | 0.49 |

**Supplementary Table S3b:** Incidence rate ratios (IRR) of serious bacterial infections among patients with inflammatory rheumatic disease one year before and after initiation of biological therapy

|  | Total sample | N infections before treatment | Observation time before treatment (years) | N infections after treatment | Observation time after treatment (years) | IRR | p-value |
| --- | --- | --- | --- | --- | --- | --- | --- |
| **Any infection** |  |  |  |  |  |  |  |
|  | 11806 | 576 | 11513 | 650 | 10634 | 1.22 (1.10-1.36) | <0.001 |
| **Any infection stratified by age** |  |  |  |  |  |  |  |
| -20 | 1080 | 82 | 1036 | 69 | 935 | 0.93 (0.68-1.27) | 0.66 |
| 21-40 | 3200 | 147 | 3122 | 138 | 2941 | 1.00 (0.80-1.24) | 0.98 |
| 41-60 | 5227 | 193 | 5132 | 263 | 4791 | 1.46 (1.22-1.75) | <0.001 |
| 61+ | 2299 | 154 | 2222 | 180 | 1968 | 1.32 (1.07-1.63) | 0.01 |
| **Site specific infections** |  |  |  |  |  |  |  |
| Bacterial infections without specified site | 11806 | 47 | 11783 | 65 | 10940 | 1.49 (1.03-2.14) | 0.03 |
| Central nervous system | 11806 | <5 | 11806 | <5 | 10967 | 3.23 (0.81-12.87) | 0.10 |
| Ear, nose, and throat | 11806 | 90 | 11759 | 105 | 10912 | 1.26 (0.96-1.65) | 0.10 |
| Respiratory tract | 11806 | 186 | 11711 | 222 | 10856 | 1.29 (1.07-1.55) | 0.01 |
| Spirochetes | 11806 | <5 | 11805 | <5 | 10967 | 1.08 (0.25-4.67) | 0.92 |
| Zoonotic bacterial infections | 11806 | <5 | 11804 | <5 | 10967 | 1.08 (0.30-3.89) | 0.91 |
| Heart | 11806 | <5 | 11805 | <5 | 10967 | 0.72 (0.16-3.30) | 0.67 |
| Skin | 11806 | 96 | 11761 | 115 | 10909 | 1.29 (0.99-1.69) | 0.06 |
| Urinary tract and genitals | 11806 | 130 | 11744 | 132 | 10903 | 1.09 (0.86-1.38) | 0.45 |
| Gastrointestinal tract | 11806 | 46 | 11784 | 61 | 10938 | 1.43 (0.98-2.08) | 0.06 |
| Eyes |  |  |  |  |  | NA |  |
| Bone and soft tissue | 11806 | 60 | 11776 | 27 | 10955 | 0.48 (0.30-0.77) | <0.01 |
